# The Incidence and Prevalence of Primary Central Nervous System (CNS) Tumours in Canada (2010–2017), and the Survival of Patients Diagnosed with CNS Tumours (2008–2017)

**DOI:** 10.3390/curroncol30040329

**Published:** 2023-04-20

**Authors:** Emily V. Walker, Yiling Zhou, Yifan Wu, Jiaqi Liu, Seth A. Climans, Faith G. Davis, Yan Yuan

**Affiliations:** 1 School of Public Health, University of Alberta, Edmonton, AB T6G 1C9, Canada; emily.walker@albertahealthservices.ca (E.V.W.); yiling8@ualberta.ca (Y.Z.); ywu8@ualberta.ca (Y.W.); jiaqileon.liu@statcan.gc.ca (J.L.); faith.davis@ualberta.ca (F.G.D.); 2 Precision Analytics, Cancer Research & Analytics, Cancer Care Alberta, Alberta Health Services, Edmonton, AB T5J 3C6, Canada; 3 Department of Oncology, Western University, London, ON N6A 5W9, Canada; seth.climans@lhsc.on.ca

**Keywords:** brain tumours, surveillance, five-year survival rate, 10-year survival curve, prevalence rate, incidence rate, unclassified tumours

## Abstract

Primary central nervous system (CNS) tumours are heterogeneous, with different treatment pathways and prognoses depending on their histological and molecular classification. Due to their anatomical location, all CNS tumours, regardless of malignancy, can be debilitating. We used vital statistics linked to Canadian Cancer Registry data to estimate the age-standardized incidence rates (ASIR), Kaplan–Meier survival rates (SR), and limited-duration prevalence proportions (PP) of 25 histology-specific CNS tumour groups that were classified based on site and histology. During 2010–2017, 45,115 patients were diagnosed with 47,085 primary CNS tumours, of which 19.0% were unclassified. The average annual ASIR was 21.48/100,000 person-years and did not vary by sex. The ASIR increased with age, particularly for meningioma, unclassified tumours, and glioblastoma. The eight-year PP was 102.1/100,000 persons (index date 1 January 2018). The most common histology was meningioma (ASIR: 5.19; PP: 31.6). The overall five-year SR among 51,310 patients diagnosed during 2008–2017 was 57.2% (95% CI: 56.8–57.7%). SRs varied by tumour behaviour, histology, and patient age, with the lowest SR among glioblastoma patients (5-year SRs ranged from 1.3–25.7%). For non-malignant tumours, the 5-year SRs ranged from 37.4–100%. We provide the most up-to-date histology-specific surveillance estimates for primary CNS tumours in Canada.

## 1. Introduction

Primary central nervous system (CNS) tumours are those originating in tissues encased by the skull and spinal column, including the brain, cranial nerves, spinal cord, cauda equina, meninges, pineal, and pituitary glands, as well as the craniopharyngeal duct [1,2]. Although they only account for ~2% of tumours diagnosed annually in Canada, the morbidity and mortality associated with primary CNS tumours are disproportionate to their incidence [3,4]. This is due to their anatomical location and capacity to inflict debilitating symptoms, such as seizures and mental impairment, regardless of tumour malignancy [5]. Routine Canadian surveillance reports provide estimates for all primary CNS tumours combined, despite the highly heterogeneous nature of these tumours with large variations in treatment and prognosis [6]. To provide more relevant information to the clinical and research communities, population surveillance estimates on specific tumour histology and behaviour categories are needed.

The Brain Tumour Surveillance Research Collaborative of Canada (BTSRC) was established in 2016 to enhance primary CNS tumour surveillance in Canada and periodically provide high-quality surveillance estimates (https://braintumourregistry.ca/ accessed on 19 December 2022) [7,8]. At the time BTRC was established, the need to improve the capture and reporting of non-malignant CNS tumours by Canadian cancer registries was highlighted, with a 2007 motion (M235) passed in the House of Commons of Canada that mandated the collection of these tumours [9]. In partnership with cancer registries in British Columbia, Alberta, Manitoba and Ontario, the BTRC identified strategies to improve the capture of non-malignant CNS tumours and/or the submission of these tumours in the respective provincial registries and subsequently in the Canadian Cancer Registry (CCR) dating as far back as 2010 in these four provinces [10].

We present the most up-to-date surveillance statistics on primary CNS tumours diagnosed in Canada between 2008 and 2017 for incidence and prevalence and between 2008 and 2018 for survival. Estimates are stratified by tumour (histology and behaviour) and/or demographic characteristics.

## 2. Methods

### 2.1. Data Sources

Each Canadian provincial/territorial cancer registry collects data on new cancer diagnoses in their respective jurisdictions, and reports them to Statistics Canada for compilation in the CCR, including diagnosis based on pathology, radiology, or clinical, among other methods [11]. The collection and use of data from these registries are governed by provincial/territorial health legislation and by the Statistics Act at the national level. We used CCR data linked with the Canadian Vital Statistics Death database and T1 Personal Master Files by Statistics Canada, which contains information on all primary tumour diagnoses among Canadian residents and their vital status between 1 January 1992, and 31 December 2017. Since cancer incidence data for Quebec was not available from 2010 onwards at the time of analysis, all estimates presented in this article exclude diagnoses from Quebec. We accessed the data through the Research Data Centre at the University of Alberta under the approval of Statistics Canada.

### 2.2. Tumour Classification

CNS tumours can be classified according to a combination of their site, histology, behaviour, and molecular features. At present, data on the molecular features of primary CNS tumours are not routinely available in the CCR. The classification system used by cancer registries in Canada is the International Classification of 2nd and 3rd edition for Topography and the 3rd edition for histology and behaviour [12]. Primary CNS tumours were defined as those occurring at the following ICD-O-3 sites: C70.0–C70.9, C71.0–71.9, C72.0–C72.9, C75.1–C75.3, and C30.0 (limited to histology codes 9522–9523). We used an updated version of ICD-O-3 that incorporates changes from the 4th edition of the WHO Classification of Tumours of the Central Nervous System [13]. Histology codes were grouped into categories based on classifications used by the Central Brain Tumour Registry of the United States (CBTRUS) [14]. Primary CNS tumours without sufficient information on pathology were labelled as “unclassified tumours”. In addition, primary CNS tumours were categorized into one of three behaviours: benign, uncertain whether benign or malignant, or malignant. For simplicity, we dichotomized tumours as either non-malignant (including benign and uncertain) or malignant.

### 2.3. Study Population

The demographic variables included in this article are sex and age group (children: 0–14 years; adolescents and young adults [AYA]: 15–39 years; adults 40–64 years; older adults: ≥65 years). Patients with missing birth and/or death dates were removed from the incidence, prevalence, and survival cohorts. In cases where dates had missing months and/or days, but not years, the missing data was imputed using an average of days. Patients diagnosed solely through death certificate or autopsy were excluded from survival analyses. For patients with the same date of diagnosis and death, the date of diagnosis was changed to one day prior to the date of death to prevent these records from being excluded from survival analysis.

### 2.4. Data Analysis

The most recent death-linked CCR data available was up to 31 December 2017. Ontario did not report primary non-malignant CNS tumours to CCR prior to 2010. We thus limited the incidence and prevalence cohort in our analysis to all primary CNS tumours diagnosed from 1 January 2010. We created a 10-year survival cohort by extending the primary CNS tumour diagnosis date back to 1 January 2008.

We used direct standardization to account for differences in the age distribution across geographic regions and over time, with the 2011 Canadian standard population as the reference when calculating incidence rates and prevalence proportions. In this article, person-based eight-year limited-duration prevalent cases are the number of patients diagnosed with primary CNS tumours between 1 January 2010 and 31 December 2017, and who were still alive as of the index date (1 January 2018). Number of prevalent cases and prevalence proportions were estimated for the age at the index date. A popular metric for reporting cancer patients’ survival is net survival, which measures the probability of surviving cancer in the absence of other causes of death [15,16]. We have provided comprehensive net survival estimates (1-, 2-, and 5-years) in our publicly available report for Canadian patients diagnosed with primary CNS tumours between 2010 and 2017 [7]. In this article, we chose to report histology-specific Kaplan–Meier (KM) survival estimates, which are more relevant clinically and complement the previously reported net survival estimates. SAS version 9.4 (SAS Institute Inc., Cary, NC, USA) and R version 4.1.2 (R Core Team, Vienna, Austria) were used to perform analyses and generate figures.

### 2.5. Multiple Primaries

All primary CNS tumours are defined according to the National Cancer Institute Surveillance, Epidemiology, and End Results (SEER) Program. Individuals may be diagnosed with more than one primary CNS tumour, which may or may not be of the same type or subtype. SEER multiple primary rules [1,17] were followed for incidence estimates; hence, the number of patients and number of tumours may differ. Only the first primary CNS tumour diagnosis was considered in the survival analysis. For prevalence estimates, only the last primary CNS tumour diagnosis was considered.

### 2.6. Disclosure Rules and Rounding

Several measures were taken to protect the confidentiality of individuals underlying the data. All frequency counts reported were randomly rounded using an unbiased random rounding scheme with a base of five. Incidence and prevalence estimates were suppressed if the category contained <5 but >0 patients. We combined categories or suppressed estimates as appropriate if stratification by specific histology groups disclosed additional categories with fewer than 5 patients. Survival estimates were suppressed if the standard error was ≥0.10, or if less than 10 patients contributed to the estimate. The study was approved by the Research Ethics Board of Alberta Cancer Committee.

## 3. Results

### 3.1. Incidence

**Figure 1:**
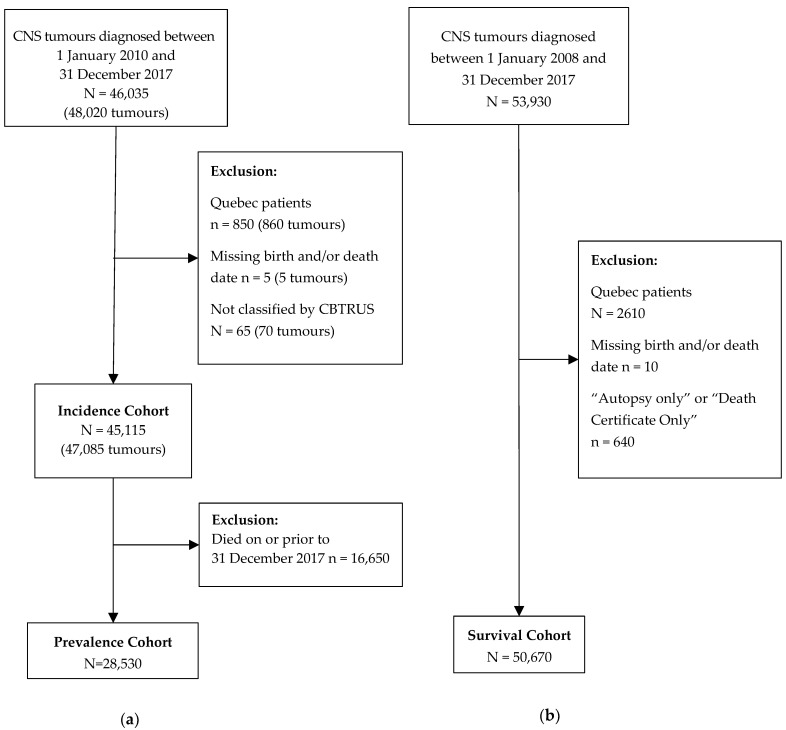
Study cohort flowchart for inclusion in the analyses. (**a**) The incidence and prevalence cohorts; (**b**) the survival cohort.

**Figure 2:**
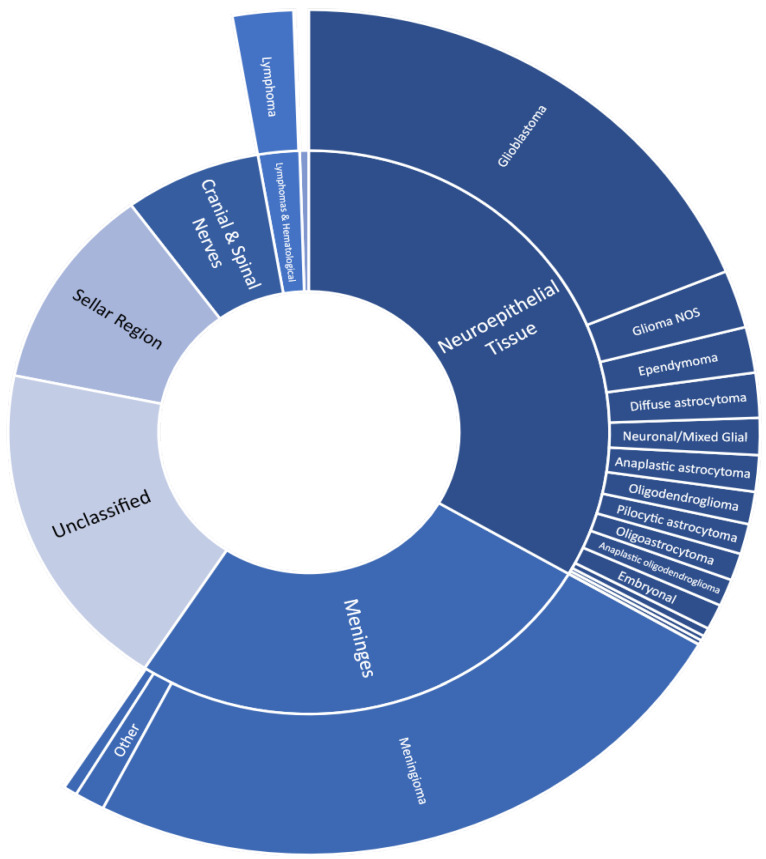
Distribution of all primary CNS tumours diagnosed among Canadians by major histology group (inner circle) and minor histology groupings (outer circle).

A total of 47,085 primary CNS tumours were diagnosed in 45,115 Canadians between 2010 and 2017 (Figure 1), corresponding to an average annual ASIR of 21.48 per 100,000 person-years (PY) (95% CI: 21.28–21.67) (Table 1). Among major histology groups, tumours of the neuroepithelial tissue had the highest annual ASIR (7.18/100,000 PY; 95% CI: 7.06–7.29) and were predominately malignant (92.9%), except for choroid plexus tumours (10.9% malignant) and neuronal and mixed neuronal-glial tumours (17.4% malignant). Glioblastoma was the most commonly diagnosed neuroepithelial tumour (ASIR: 4.01/100,000 PY; 95% CI: 3.93–4.09), and meningioma was the most commonly diagnosed histology overall (ASIR: 5.18/100,000 PY; 95% CI: 5.09–5.28) (Table 1; Figure 2). Meningioma and other tumours of the meninges were primarily non-malignant (3%).

#### 3.1.1. Incidence Stratified by Sex

There was no noticeable difference in overall incidence by sex: the ASIR for females was slightly higher than that of males (females: ASIR: 21.87/100,000 PY (95% CI: 21.60–22.15); males: ASIR: 21.11/100,000 PY (95% CI: 20.83–21.39)). However, there were differences in the frequencies of some major and specific histology groups between the sexes. The most common major histology group among males was tumours of the neuroepithelial tissue (ASIR: 8.56/100,000 PY; 95% CI: 8.38–8.74), whereas, for females, it was tumours of the meninges (ASIR: 7.25/100,000 PY; 95% CI: 7.09–7.41). The most common specific histology group among males was glioblastoma (ASIR: 4.91/100,000 PY; 95% CI: 4.81–5.08), whereas, for females, it was meningioma (ASIR: 6.91/100,000 PY; 95% CI: 6.76–7.06). Conversely, females and males had similar ASIRs for the following histological subtypes: choroid plexus tumours, tumours of the pineal region, tumours of the cranial and spinal nerves, mesenchymal tumours, primary melanocytic lesions, and other hematopoietic neoplasms. Males had a higher percentage of malignant tumours compared to females (43.7% vs. 28.8%).

#### 3.1.2. Incidence Stratified by Age

Overall, the ASIRs for primary CNS tumours increased with age. The ASIRs in children, AYA, adults, and older adults were 5.30 (95% CI: 5.06–5.54), 9.00 (95% CI: 8.78–9.22), 25.88 (95% CI: 25.52–26.25), and 58.16 (95% CI: 57.33–58.99) per 100,000 PY, respectively (Table 2). The most frequently diagnosed major histology group in all age groups was tumours of the neuroepithelial tissue. The corresponding ASIRs were 3.70 (95% CI: 3.50–3.91), 3.40 (95% CI: 3.26–3.53), 8.48 (95% CI: 8.27–8.69), and 16.74 (95% CI: 16.30–17.20) per 100,000 PY in the four age groups, respectively. The steepest increases in incidence with age were observed for tumours of the neuroepithelial tissue (predominantly glioblastomas), tumours of the meninges (predominantly meningiomas), and unclassified tumours. Among older adults, unclassified tumours also had a high ASIR of 16.02/100,000 (95% CI: 15.59–16.46), which was similar to that of neuroepithelial tumours. The proportion of neuroepithelial tumours that were malignant either remained stable or increased with age, depending on the histology. Exceptions included ependymal tumours and tumours of the pineal region, where malignant behaviours were more common among children than in other age groups (90.5% and 100.0% malignant for each histology, respectively). Tumours of the cranial and spinal nerves, tumours of the meninges, germ cell tumours, cysts and heterotopias, and tumours of the sellar region also exhibited decreased percent malignancy with increased age.

### 3.2. Survival

**Figure 3:**
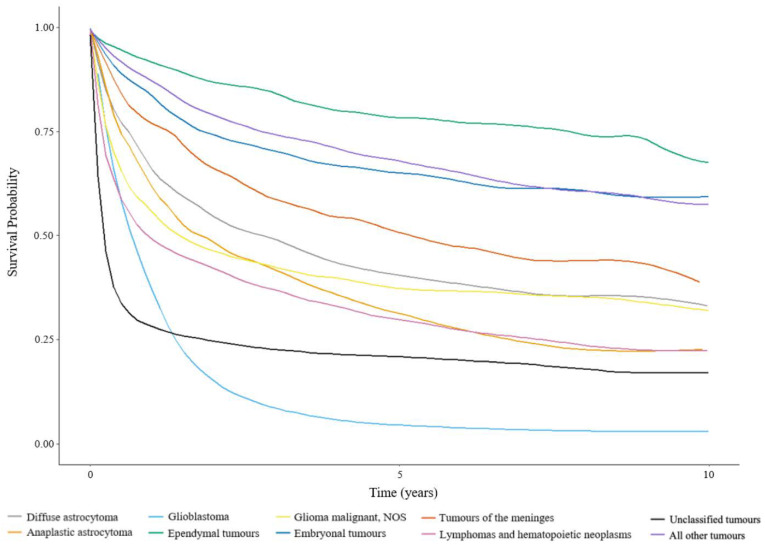
Kaplan–Meier survival curves for primary malignant CNS tumour patients diagnosed between 2008 and 2017 in Canada (excluding Quebec) stratified by selected histology. “All other tumours” include the following: pilocytic astrocytoma, unique astrocytoma variants, oligodendroglioma, anaplastic oligodendroglioma, oligoastrocytic tumours, choroid plexus tumours, neuronal and mixed neuronal-glial tumours, tumours of the cranial and spinal nerves, germ cell tumours, cysts and heterotopias, tumours of the sellar region.

**Figure 4:**
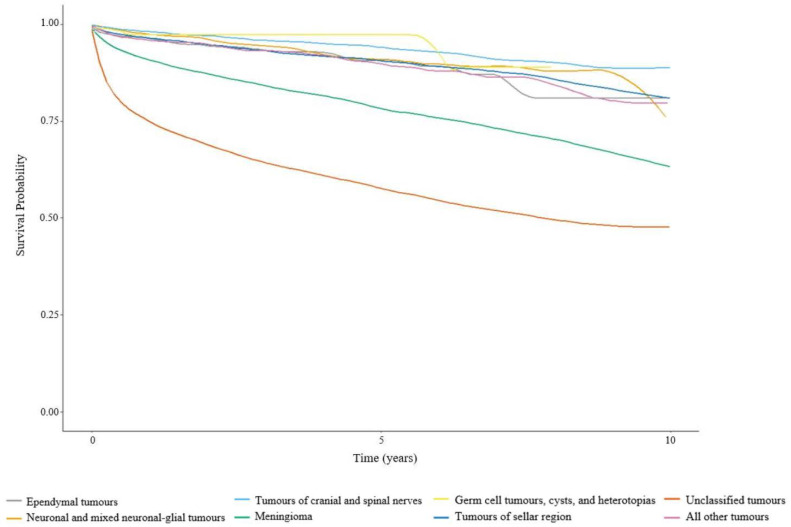
Kaplan–Meier survival curves for primary non-malignant CNS tumour patients diagnosed between 2008 and 2017 in Canada (excluding Quebec), stratified by selected histology. “All other tumours” include the following: unique astrocytoma variants, choroid plexus tumours, mesenchymal tumours, primary melanocytic lesions, other neoplasms related to the meninges.

There were 50,670 primary CNS tumour patients diagnosed between 2008 and 2017 that were included in the survival analyses (Figure 1). Figure 3 and Figure 4 present KM survival curves for selected malignant and non-malignant CNS tumours, respectively. Patients with non-malignant tumours had better overall survival than those with malignant tumours. Among malignant tumours, ependymal and embryonal tumours had favourable long-term prognoses, as both histological subtypes had median survival times (MSTs) of >10 years (Figure 3). Conversely, glioblastomas and unclassified tumours had unfavourable prognoses, with MSTs of <2.5 years. The KM survival curves for glioblastoma, unclassified tumours, lymphomas and hematopoietic neoplasms, anaplastic astrocytoma, malignant glioma (NOS), and diffuse astrocytoma showed a large decline in survival within the first few years of diagnosis. For tumours of the meninges, embryonal tumours, ependymal tumours, and all other tumours, a gradual, steady decline in survival was observed over time.

Among those with non-malignant tumours, patients with unclassified tumours had the poorest long-term survival, with a pronounced decrease in survival rate within the first year after diagnosis (Figure 4). Patients with meningioma also had poor long-term survival relative to those with other non-malignant tumours, but with a survival curve that decreased steadily with time. Patients with germ cell tumours, cysts, and heterotopias exhibited stable survival until around five years after diagnosis, after which a decline in survival was observed. Similarly, survival for patients with ependymal tumours and neuronal and mixed neuronal-glial tumours remained relatively stable until around seven and nine years after diagnosis, respectively, after which there was a notable decline in survival for each histology.

#### 3.2.1. Survival Stratified by Age for Malignant Tumours

Estimated 5-year SRs and MSTs for patients with primary malignant CNS tumours stratified by age group and histology are presented in Table 3. The MST ranged from 11 months to >10 years in children, 2 to >10 years in AYA, 1 to >10 years in adults, and 2 months to 9 years in older adults. Overall 5-year SRs decreased with age from 72.9% (95% CI: 70.5–75.3%) in children, to 68.4% (95% CI: 66.5–70.4%) in AYA, to 24.3% (95% CI: 23.3–25.4%) in adults, to 5.9% (95% CI: 5.4–6.6%) in older adults. While survival varied greatly by histology, younger patients (children and AYA) consistently exhibited better survival compared to older patients with the same histology. Children had higher 5-year SR estimates for pilocytic astrocytoma compared to AYA. Conversely, AYA had higher 5-year SR estimates for anaplastic astrocytoma, glioblastoma, ependymal tumours, malignant gliomas (NOS), and tumours of the meninges compared to children. Survival for patients with anaplastic astrocytoma was notably higher in AYA (5-year SR: 63.3%; 95% CI: 56.4–71.1%) compared to children (5-year SR: 25.7%; 95% CI: 12.6–52.3%). From AYA onwards, 5-year SRs decreased substantially with increased age. The largest declines in 5-year SRs with age (AYA to older adults) were observed for diffuse astrocytoma, followed by oligoastrocytic tumours, unclassified tumours, and malignant gliomas (NOS) in descending order. However, adults and older adults had similar 5-year SRs for unique astrocytoma variants (22.7%; 95% CI: 11.7–43.7% versus 16.1%; 95% CI: 6.9–37.8%) and mesenchymal tumours (51.4%; 95% CI: 37.3–70.7% versus 44.1%; 95% CI: 25–77.8%). Among all major and specific histology groups, as well as patients with glioblastomas, had the lowest 5-year SRs across all age groups, which ranged from 1.3% in older adults (95% CI: 0.9–1.8%) to 25.7% in AYA (95% CI: 21.2–31.1%), whereas pilocytic astrocytoma had the highest 5-year SRs across all age groups except for in older adults, which ranged from 88.0% in adults (95% CI: 80.3–96.4%) to 98.9% in children (95% CI: 97.9–100.0%). For older adults, ependymal tumours had the best 5-year SR of 56.9% (95% CI: 46.5–69.7%).

#### 3.2.2. Survival Stratified by Age for Non-Malignant Tumours

Estimated 5-year SRs and MSTs for patients with primary non-malignant CNS tumours stratified by age group and histology are presented in Table 4. The MST was >10 years in children and AYA, ranged from 9.43 to >10 years in adults, and ranged from 2.58 to >10 years in older adults (Table 4). Overall, 5-year SRs were relatively high for children (96.3%; 95% CI: 94.7–97.9%), AYA (96.9%; 95% CI: 96.3–97.5%), and adults (89.8%; 95% CI: 89.2–90.4%). Older adults had a notably lower overall 5-year SR of 57.9% (95% CI: 56.9–59.0%) and for unclassified tumours (5-year SR: 37.4%; 95% CI: 35.6–39.4%). Similar to malignant tumours, survival was also the highest among children and AYA for non-malignant tumours. Children and AYA had similar 5-year SRs for all major and specific histology groups, approaching or achieving 100%. Tumours of the cranial and spinal nerves had consistently high 5-year survival estimates across all age groups (children 5-year SR: 100%; AYA 5-year SR: 98.5%, 95% CI: 97.4–99.7%; adults 5-year SR: 96.9%, 95% CI: 95.9–97.8%; older adults 5-year SR: 83.2%, 95% CI: 80.2–86.3%).

### 3.3. Prevalence Stratified by Age and Tumour Behaviour

**Figure 5:**
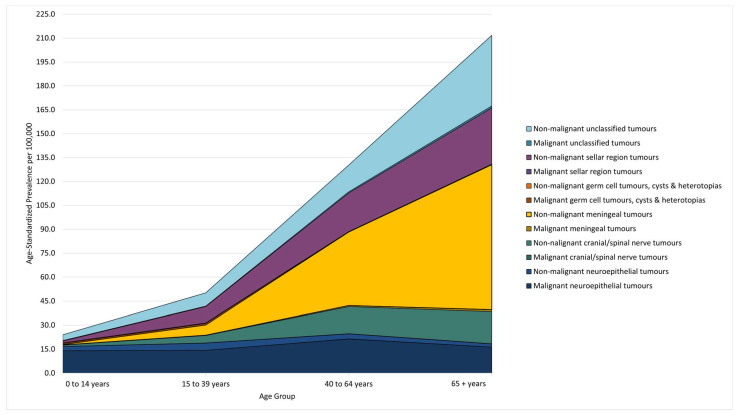
Age-adjusted prevalence proportion per 100,000 of primary malignant CNS tumour patients diagnosed between 2008 and 2017 in Canada (excluding Quebec), stratified by selected histology and behaviour.

There were 28,530 Canadians who were diagnosed with a primary CNS tumour between 2010 and 2017 and who were alive as of 1 January 2018 (Figure 1). Among these patients, 5660 had malignant tumours (19.7%) and 23,075 had non-malignant tumours (80.3%). For malignant tumours, an increased PP with increased age was observed for the following histological subtypes: glioblastoma, meningioma, lymphoma and unclassified tumours (Table 5; Figure 5). Conversely, a decreased PP with increased age was observed for pilocytic astrocytoma and malignant embryonal tumours. For most non-malignant neuroepithelial tumour subtypes, the PP increased from children to AYA and adults and declined in older adults (Table 6; Figure 5). Non-malignant tumours of the cranial and spinal nerves, meningiomas, tumours of the sellar region, and unclassified tumours had greater increases in the PP with age relative to the remaining histological subtypes, which maintained low, stable PP estimates across age categories (Figure 5).

### 3.4. Unclassified Tumours

Although Ontario had the highest proportion of unclassified primary malignant CNS tumours in Canada from 2008 to 2016 (17.4% to 8.0%), this proportion decreased over time and is presented in Supplementary Table S1. In 2017, Manitoba and New Brunswick surpassed Ontario as the two provinces with the highest proportions of unclassified malignant CNS tumours in Canada (9.5% and 7.1%, respectively). Additionally, New Brunswick experienced a large increase in the proportion of unclassified malignant tumours between 2008 and 2009 (from 8.3% to 14.3%). Ontario also had the highest proportion of unclassified primary non-malignant CNS tumours between 2010 and 2017 (ranging from 34.1% to 42.7%), compared to <10% in all other provinces. Data on unclassified tumours was unavailable for all territories. In addition, the Ontario Cancer Registry did not report non-malignant tumours prior to 2010; its effect can be seen in Supplementary Figure S1.

## 4. Discussion

We presented population-based surveillance of CNS tumours in Canada from 2010–2017 (excluding Quebec). The average annual age-standardized incidence rate (ASIR) per 100,000 for all primary CNS tumours in Canada (excluding Quebec) was 21.48 (95% CI: 21.28–21.67). Although the ASIR for all CNS tumours combined was similar for males (20.73 per 100,000, 95% CI: 20.38–21.07) and females (21.40, 95% CI: 21.07–21.74), differences existed by histology. Men had higher rates for glioblastoma, while females had higher rates for meningioma. The ASIR for all CNS tumours combined significantly increased with age from 5.30 per 100,000 (95% CI: 5.06–5.54) in children (age 0 to 14 years), to 9.00 (95% CI: 8.78–9.22) in adolescents and young adults (age 15–39 years), to 25.88 (95% CI: 25.52–26.25) in adults (age 40–64 years), to 58.16 per 100,000 (95% CI: 57.33–58.99) in older adults (65+). Interestingly, the proportion of incident tumour diagnoses that were malignant was consistent across all adolescent and adult age groups at ~34%, but approximately double among those aged 0–14 years (67%). Of classifiable histological subtypes, 8 were associated with a median survival time of more than 8 years. The shortest median survival was among patients with glioblastoma (8 months) or lymphoma (12 months). Younger age was associated with longer survival time. However, the AYA age category had the longest survival time for glioblastoma and anaplastic astrocytoma. At the index date of 1 January 2018, there were an estimated 28,530 patients living with a primary CNS tumour in Canada for an eight-year limited-duration prevalence proportion (PP) of 102.1 per 100,000 persons. Of these, 80.3% were non-malignant, which is consistent with the longer survival time among those diagnosed with non-malignant tumours.

Patients with unclassified tumours consistently had poor survival outcomes, which were second only to glioblastoma patients for malignant behaviours. It is difficult to interpret the meaning of this finding, given the potential heterogeneity in tumours included in these unclassified categories. Within Canada, the highest proportion of unclassified tumours was in Ontario, which was predominantly driven by non-malignant unclassified tumours with an incidence proportion approximately 7.6 times that of other provinces included in the analysis (Supplementary Table S1). Conversely, the proportion of malignant unclassified tumours was consistent across provinces (Supplementary Table S1). This indicates that, while Ontario appeared to capture the greatest proportion of expected non-malignant tumours relative to other provinces, a large proportion of those tumours were not classified by histology. Therefore, in addition to improving the overall capture of non-malignant tumours in provincial/territorial cancer registries, additional work is needed to ensure consistent and complete classification of these tumours across Canada to better understand the patterns of diagnosis and prognosis for patients diagnosed with CNS tumours in this country.

Overall, the findings are consistent with other regions. The overall rate of CNS tumours in Canada is lower than that of the United States (U.S: 23.79/100,000; Canada: 21.48/100,000) [18]. In addition to slight variations in the age distribution of the underlying populations, which was not accounted for in this comparison, this difference was largely driven by non-malignant tumours, which were lower than expected in Canada due to incomplete capture in provincial/territorial cancer registries [19]. While this capture has vastly improved since 2009, there is more work to be done to ensure that each of these tumours is routinely counted. In terms of histology, the largest difference between Canada and the United States was among tumours of the meninges. This further supports the interpretation of differences being largely driven by the under-reporting of non-malignant tumours in Canada, as these tumours are predominantly non-malignant. The second largest difference was for unclassified tumours, with a higher rate of these tumours in Canada. This comparison flags an additional issue in the Canadian data that needs to be addressed. In a review of the global burden of primary malignant CNS tumours, Patel et al. [20] highlighted Nordic countries as having some of the highest age-standardized rates, ranging from 13·52/100,000 in Finland to 20·76/100,000 in Iceland. As with the present analysis, the findings from this review highlight glioblastoma as one of the most common histological subtypes to be diagnosed [20,21].

European 5-year relative survival estimates for primary malignant neuroepithelial tumours from the EUROCARE-5 study showed survival estimates of 24.6% for Norway, 17.9% for Ireland and UK, 25.1% for Germany, 18.5% for France, and 20.3% for Europe as a whole [22]. Glioblastoma 5-year relative survival was reported to be 3.4% in the UK [23], 6.3% overall in Europe [22], and the crude survival was estimated to be 4.9% in the present analysis. However, these comparisons must be interpreted with caution because of differences in methods of data capture, calculating survival, and grouping of CNS tumours into histological categories. The estimated median survival in this report was lowest for those with glioblastoma (8 months). Ostrom et al. [18] estimated the same median survival time for glioblastoma patients in the United States. Population-based studies in England from 2007–2011 reported a median survival length of 6.1 months for glioblastoma patients. Differences in estimates across regions may reflect the time periods of the analysis. Changes in CNS tumour classification in recent years could also have contributed to the observed differences.

The estimates presented in this paper represent broader categories of histological subtypes than may be relevant clinically. This is because primary CNS tumours are rare, and the number of diagnoses is often too low to permit reporting on smaller categories of tumours while adhering to reporting guidelines set by Statistics Canada to ensure the privacy of those represented by these numbers. Additionally, data on molecular markers are not currently available through the CCR. Therefore, tumour classifications do not strictly include molecular subtypes, although this information may have been included in the initial diagnosis and used to inform treatment decisions. There is incomplete correspondence between the ICD-O-3 diagnoses used in our analysis and the 2021 World Health Organization classification of tumours of the CNS [24]. This limits the clinical applicability of our analysis. It has been suggested that switching from ICD-O-3 to ICD-11 might better capture the evolving molecular diagnoses of CNS tumours [24]. Finally, while this analysis yielded the most complete Canadian data on primary CNS tumours to date, it remains incomplete due to a lack of data from the province of Quebec in the CCR past diagnosis year 2010. We anticipate including Quebec in subsequent analyses, as Quebec data from year 2010–2017 will be made available to the CCR in 2023.

## 5. Conclusions

Overall, the findings were consistent with the expected frequency and distribution of primary CNS tumours in Canada and comparable to those reported in US and some European countries. These findings reflect the improvements made by provinces in collaboration with the BTRC in capturing non-malignant CNS tumours in their respective registries. However, there remains evidence of incomplete capture to varying degrees across provinces. Further, the high frequency of unclassified tumours indicates the need for further efforts to improve the consistency and completeness of information across provinces. The BTRC continues to collaborate with provincial cancer registries to identify strategies for addressing the issues highlighted in this manuscript. Currently these are the most up-to-date histology-specific brain tumour surveillance estimates available in Canada.

## Figures and Tables

**Table 1:** Average number of cases per year ($\overset{-}{X}$), percent malignant ^e^ (% M), average annual age-standardized incidence rates ^a^ (ASIRs, per 100,000 person-years) and associated 95% confidence intervals (CIs) for all primary central nervous system (CNS) tumours diagnosed between 2010–2017 in Canada (excluding Quebec) by histology and sex.

	Total				Male				Female			
Histology Group ^b^ (Major/Specific)	$\overset{-}{X}$	% M ^e^	ASIR	95% CI	$\overset{-}{X}$	% M ^e^	ASIR	95% CI	$\overset{-}{X}$	% M ^e^	ASIR	95% CI
**Tumours of neuroepithelial tissue**	1967	92.9	7.18	7.06–7.29	1137	93.1	8.56	8.38–8.74	830	92.7	5.90	5.76–6.04
Pilocytic astrocytoma	68	100.0	0.25	0.23–0.27	34	100.0	0.25	0.22–0.28	33	100.0	0.25	0.22–0.28
Diffuse astrocytoma	101	100.0	0.37	0.34–0.40	59	100.0	0.45	0.41–0.49	41	100.0	0.30	0.27–0.33
Anaplastic astrocytoma	82	100.0	0.30	0.28–0.32	44	100.0	0.33	0.29–0.36	38	100.0	0.28	0.25–0.31
Unique astrocytoma variants	19	70.4	0.07	0.06–0.08	12	68.9	0.09	0.07–0.11	8	73.0	0.05	0.04–0.07
Glioblastoma	1106	100.0	4.01	3.93–4.09	651	100.0	4.95	4.81–5.08	455	100.0	3.16	3.06–3.26
Oligodendroglioma	73	100.0	0.27	0.25–0.29	42	100.0	0.32	0.28–0.35	31	100.0	0.22	0.19–0.25
Anaplastic oligodendroglioma	61	100.0	0.23	0.21–0.25	36	100.0	0.27	0.24–0.30	26	100.0	0.19	0.16–0.21
Oligoastrocytic tumours	61	100.0	0.23	0.21–0.25	35	100.0	0.26	0.23–0.29	27	100.0	0.20	0.17–0.22
Ependymal tumours	103	58.7	0.38	0.35–0.40	59	57.1	0.44	0.40–0.48	43	60.6	0.32	0.28–0.35
Glioma malignant, NOS ^c^	130	100.0	0.48	0.45–0.51	70	100.0	0.53	0.49–0.58	61	100.0	0.43	0.39–0.47
Choroid plexus tumours	11	10.9	0.04	0.03–0.05	5	11.1	0.04	0.03–0.05	6	10.8	0.05	0.03–0.06
Neuronal & mixed neuronal-glial tumours	83	17.4	0.31	0.29–0.33	49	18.5	0.36	0.32–0.39	35	15.8	0.26	0.23–0.29
Tumours of the pineal region	10	67.3	0.04	0.03–0.05	5	64.3	0.04	0.03–0.05	5	70.8	0.04	0.03–0.05
Embryonal tumours	57	97.7	0.21	0.19–0.23	35	98.4	0.25	0.22–0.28	22	96.6	0.16	0.14–0.19
Other neuroepithelial tumours ^d^	1	80.0	0.01	0.00–0.01	1 *	66.7	-	-	1 *	-	-	-
**Tumours of cranial & spinal nerves**	433	0.9	1.59	1.54–1.65	213	0.8	1.60	1.52–1.67	220	1.0	1.59	1.52–1.67
**Tumours of meninges**	1518	3.0	5.54	5.44–5.64	483	4.7	3.72	3.61–3.84	1036	2.3	7.25	7.09–7.41
Meningioma	1422	2.1	5.18	5.09–5.28	433	3.0	3.35	3.24–3.47	989	1.7	6.91	6.76–7.06
Mesenchymal tumours	29	35.5	0.11	0.09–0.12	14	42.5	0.10	0.08–0.12	16	29.1	0.11	0.09–0.13
Primary melanocytic lesions	4	63.6	0.01	0.01–0.02	2	66.7	0.01	0.01–0.02	1	60.0	0.01	0.01–0.02
Other neoplasms related to the meninges	64	3.7	0.23	0.21–0.26	34	4.3	0.25	0.22–0.29	29	3.1	0.22	0.19–0.25
**Lymphomas & hematopoietic neoplasms**	130	99.8	0.47	0.44–0.50	73	100.0	0.55	0.51–0.60	58	99.6	0.40	0.36–0.43
Lymphoma	128	100.0	0.46	0.43–0.49	71	100.0	0.54	0.50–0.59	56	100.0	0.39	0.35–0.42
Other hematopoietic neoplasms	3	92.9	0.01	0.01–0.02	2	100.0	0.01	0.01–0.02	2	90.0	0.01	0.01–0.02
**Germ cell tumours, cysts, & heterotopias**	29	62.7	0.11	0.09–0.12	21	71.4	0.15	0.13–0.18	8	41.3	0.06	0.04–0.07
**Tumours of sellar region**	689	0.4	2.53	2.46–2.59	353	0.6	2.67	2.57–2.77	336	0.3	2.43	2.34–2.52
**Unclassified tumours**	1121	-	4.07	3.98–4.15	484	-	3.86	3.74–3.99	637	-	4.25	4.14–4.37
Total	5886	35.7	21.48	21.28–21.67	2762	43.7	21.11	20.83–21.39	3124	28.8	21.87	21.60–22.15

Notes: columns and rows may not sum to totals due to rounding. ^a^ Rates are age-standardized to the 2011 Canadian standard population. ^b^ Defined as per the Central Brain Tumour Registry of the United States. ^c^ NOS = not otherwise specified. ^d^ Histology groups that have combined average case counts across multiple groups in each row indicated by *. ^e^ % M is estimated from 2010–2015 British Columbia, Alberta, Manitoba, and Ontario combined CNS tumour incidence; % M for unclassified tumours is unavailable.

**Table 2:** Average annual cases ($\overset{-}{X}$), percent malignant ^e^ (% M), average annual age-standardized incidence rates ^a^ (ASIRs, per 100,000 person-years) and associated 95% confidence intervals (CIs) for all primary CNS tumours diagnosed between 2010–2017 in Canada (excluding Quebec) by histology and age group.

	Age Group (Years)															
	0 to 14				15 to 39					40 to 64			65+			
Histology Group ^b^ (Major/Specific)	$\overset{-}{X}$	% M ^e^	ASIR	95% CI	$\overset{-}{X}$	% M ^e^	ASIR	95% CI	$\overset{-}{X}$	% M ^e^	ASIR	95% CI	$\overset{-}{X}$	% M ^e^	ASIR	95% CI
**Tumours of neuroepithelial tissue**	166	85.2	3.70	3.50–3.91	310	81.9	3.40	3.26–3.53	809	96.3	8.48	8.27–8.69	682	96.3	16.74	16.30–17.20
Pilocytic astrocytoma	38	100.0	0.84	0.75–0.94	20	100.0	0.23	0.19–0.26	8	100.0	0.09	0.07–0.11	1	100.0	0.03	0.02–0.06
Diffuse astrocytoma	9	100.0	0.19	0.15–0.24	30	100.0	0.33	0.29–0.37	41	100.0	0.43	0.38–0.48	21	100.0	0.52	0.45–0.61
Anaplastic astrocytoma	3	100.0	0.06	0.04–0.09	25	100.0	0.28	0.24–0.32	34	100.0	0.35	0.31–0.40	21	100.0	0.50	0.43–0.58
Unique astrocytoma variants	4	40.0	0.09	0.06–0.13	8	62.5	0.08	0.06–0.11	4	94.7	0.05	0.04–0.07	3	94.7	0.08	0.05–0.12
Glioblastoma	8	100.0	0.18	0.14–0.23	53	100.0	0.58	0.52–0.64	506	100.0	5.28	5.12–5.45	539	100.0	13.25	12.85–13.65
Oligodendroglioma ^d^	29 *	-	-	-	29 *	100.0	0.31	0.27–0.36	38	100.0	0.40	0.36–0.45	6	100.0	0.13	0.10–0.18
Anaplastic oligodendroglioma ^d^	15 *	-	-	-	15 *	100.0	0.16	0.14–0.20	36	100.0	0.38	0.34–0.43	10	100.0	0.24	0.19–0.30
Oligoastrocytic tumours	1	-	0.01	0.00–0.03	22	100.0	0.24	0.20–0.27	31	100.0	0.33	0.29–0.37	8	100.0	0.20	0.16–0.26
Ependymal tumours	16	90.5	0.36	0.30–0.43	28	58.4	0.30	0.26–0.34	42	50.5	0.44	0.40–0.49	17	50.5	0.41	0.34–0.49
Glioma malignant, NOS ^c^	26	100.0	0.59	0.51–0.67	26	100.0	0.28	0.24–0.32	34	100.0	0.36	0.32–0.41	44	100.0	1.09	0.98–1.21
Choroid plexus tumours	5	19.4	0.11	0.08–0.15	2	7.7	0.02	0.01–0.03	3	-	0.03	0.02–0.05	1	-	0.02	0.01–0.05
Neuronal & mixed neuronal-glial tumours	16	3.4	0.37	0.31–0.43	38	7.9	0.42	0.37–0.47	21	41.7	0.23	0.19–0.26	8	41.7	0.19	0.14–0.24
Tumours of the pineal region	3	100.0	0.06	0.04–0.09	3	71.4	0.03	0.02–0.04	4	45.8	0.04	0.03–0.06	1	45.8	0.03	0.01–0.05
Embryonal tumours	36	99.0	0.82	0.73–0.92	13	97.1	0.14	0.11–0.17	6	92.1	0.06	0.04–0.08	2	92.1	0.04	0.02–0.07
Other neuroepithelial tumours ^d^	1 *	66.7	-	-	1 *	.	-	-	1 *	-	-	-	1 *	-	-	-
**Tumours of cranial & spinal nerves**	8	5.9	0.18	0.14–0.23	88	1.6	0.96	0.89–1.03	237	0.6	2.50	2.39–2.62	99	0.6	2.43	2.26–2.60
**Tumours of meninges**	6	48.6	0.13	0.10–0.18	126	3.7	1.37	1.28–1.46	724	2.7	7.61	7.41–7.80	663	2.7	16.32	15.88–16.77
Meningioma	3	12.5	0.06	0.04–0.09	103	2.1	1.11	1.04–1.19	675	2.1	7.09	6.90–7.28	642	2.1	15.81	15.38–16.25
Mesenchymal tumours	2	83.3	0.04	0.02–0.07	4	47.6	0.05	0.03–0.06	16	29.3	0.17	0.14–0.20	8	29.3	0.17	0.13–0.22
Primary melanocytic lesions ^d^	1	100.0	0.02	0.01–0.04	1	40.0	0.01	0.00–0.02	2 *	58.3	0.02	0.01–0.03	2 *	58.3	-	-
Other neoplasms related to the meninges	1	25.0	0.02	0.01–0.04	18	1.0	0.20	0.17–0.23	31	4.5	0.33	0.29–0.37	14	4.5	0.33	0.27–0.40
**Lymphomas & hematopoietic neoplasms**	1	100.0	0.02	0.01–0.04	7	100.0	0.08	0.06–0.10	49	99.8	0.52	0.47–0.57	73	99.8	1.78	1.64–1.93
Lymphoma	1	100.0	0.01	0.00–0.03	6	100.0	0.07	0.05–0.09	48	100.0	0.51	0.46–0.56	71	100.0	1.76	1.62–1.91
Other hematopoietic neoplasms ^d^	1 *	-	-	-	1 *	-	-	-	1	91.7	0.01	0.01–0.02	1	91.7	0.02	0.01–0.04
**Germ cell tumours, cysts, & heterotopias**	12	72.7	0.26	0.21–0.32	13	68.3	0.15	0.12–0.18	3	14.3	0.03	0.02–0.05	1	14.3	0.02	0.01–0.04
**Tumours of sellar region**	13	3.4	0.27	0.22–0.33	153	0.2	1.67	1.58–1.77	327	0.4	3.44	3.31–3.58	198	0.4	4.84	4.60–5.09
**Unclassified tumours**	33	-	0.73	0.65–0.83	126	-	1.38	1.29–1.47	314	-	3.30	3.17–3.43	648	-	16.02	15.59–16.46
Total	237	67.1	5.30	5.06–5.54	823	34.2	9.00	8.78–9.22	2464	34.4	25.88	25.52–26.25	2363	34.4	58.16	57.33–58.99

Notes: columns and rows may not sum to totals due to rounding. ^a^ Rates are age-standardized to the 2011 Canadian standard population. ^b^ Defined as per the Central Brain Tumour Registry of the United States. ^c^ NOS = not otherwise specified. ^d^ Histology groups that have combined average case counts across multiple groups in each row indicated by *. ^e^ % M is estimated from 2010–2015 British Columbia, Alberta, Manitoba, and Ontario combined CNS tumour incidence; % M for age groups 40–64 and 65+ are combined; % M for unclassified tumours is unavailable.

**Table 3:** Median survival times (MSTs), five-year survival rates (5-year SRs), and associated 95% confidence intervals (CIs) for patients with primary malignant CNS tumours diagnosed between 2010–2017 in Canada (excluding Quebec) by histology and age group.

	Age Group (Years)											
	0 to 14			15 to 39			40 to 64			65+		
Histology Group ^a^ (Major/Specific)	MST ^d^	5-Year SR ^e^	95% CI	MST ^d^	5-Year SR ^e^	95% CI	MST ^d^	5-Year SR ^e^	95% CI	MST ^d^	5-Year SR ^e^	95% CI
**Tumours of neuroepithelial tissue**	>120	72.4	69.9–75.0	>120	67.6	65.5–69.8	15.8	21.1	20.0–22.2	4.9	3.7	3.2–4.3
Pilocytic astrocytoma ^c^	>120	98.9	97.9–100.0	>120	93.2	89.5–97.0	>120	88.0	80.3–96.4	-	-	-
Diffuse astrocytoma	>120	72.7	63.5–83.3	>120	74.4	68.7–80.6	27.9	33.1	28.3–38.7	4.9	3.8	1.9–7.8
Anaplastic astrocytoma	11.0	25.7	12.6–52.3	81.7	63.3	56.4–71.1	18.6	23.0	17.9–29.7	5.1	3.2	0.6–16.0
Unique astrocytoma variants	>120	63.0	44.4–89.3	>120	67.4	53.7–84.8	14.3	22.7	11.7–43.7	3.9	16.1	6.9–37.8
Glioblastoma	11.3	14.3	7.0–28.9	25.3	25.7	21.2–31.1	12.2	5.9	5.1–6.7	4.8	1.3	0.9–1.8
Oligodendroglioma ^c^	-	-	-	>120	88.5	84.0–93.4	>120	75.8	71.0–80.9	14.1	30.5	19.2–48.4
Anaplastic oligodendroglioma ^c^	-	-	-	>120	73.1	65.0–82.2	64.5	52.2	46.3–58.8	11.2	14.8	8.7–25.2
Oligoastrocytic tumours ^c^	-	-	-	>120	76.3	70.7–82.4	51.1	44.8	39.4–51.0	9.1	7.6	3.4–17.0
Ependymal tumours	>120	79.2	71.7–87.3	>120	87.5	81.5–94.1	>120	81.2	75.1–87.9	108.9	56.9	46.5–69.7
Glioma malignant, NOS ^b^	>120	59.1	53.1–65.8	>120	71.7	65.8–78.0	29.3	35.3	29.8–41.7	3.2	6.3	4.2–9.4
Choroid plexus tumours ^c^	-	-	-	-	-	-	-	-	-	-	-	-
Neuronal & mixed neuronal-glial tumours ^c^	-	-	-	>120	86.0	72.3–100.0	>120	57.0	45.6–71.2	20.7	31.8	18.0–56.1
Tumours of the pineal region ^c^	44.5	48.4	30.3–77.2	-	-	-	-	-	-	-	-	-
Embryonal tumours ^c^	>120	68.0	62.8–73.5	>120	65.6	56.9–75.6	68.7	57.3	42.7–76.7	-	-	-
Other neuroepithelial tumours ^c^	-	-	-	-	-	-	-	-	-	-	-	-
**Tumours of cranial & spinal nerves ^c^**	-	-	-	-	-	-	-	-	-	-	-	-
**Tumours of meninges**	44.6	41.3	25.5–66.9	>120	67.9	56.4–81.8	81.4	51.8	44.0–61.0	34.7	41.8	33.7–51.8
Meningioma ^c^	-	-	-	>120	69.9	53.4–91.4	>120	55.3	45.3–67.5	35.2	40.3	31.3–52.0
Mesenchymal tumours ^c^	-	-	-	>120	70.4	52.7–93.9	>120	51.4	37.3–70.7	28.5	44.1	25–77.8
Primary melanocytic lesions ^c^	-	-	-	-	-	-	-	-	-	-	-	-
Other neoplasms related to the meninges ^c^	-	-	-	-	-	-	64.6	52.1	32.3–84.2	-	-	-
**Lymphomas & hematopoietic neoplasms ^c^**	-	-	-	>120	57.2	45.5–71.9	46.3	44.4	39.4–50.0	5.1	16.1	13.0–19.8
Lymphoma ^c^	-	-	-	>120	58.2	46.3–73.2	46.3	44.4	39.3–50.1	5.1	16.1	13.1–19.9
Other hematopoietic neoplasms ^c^	-	-	-	-	-	-	-	-	-	-	-	-
**Germ cell tumours, cysts, & heterotopias ^c^**	>120	88.7	82.0–96.0	>120	90.2	83.9–96.9	-	-	-	-	-	-
**Tumours of sellar region ^c^**	-	-	-	-	-	-	-	-	-	-	-	-
**Unclassified tumours**	>120	76.8	64.5–91.4	>120	73.6	65.0–83.3	37.5	47.9	42.7–53.8	1.9	6.4	5.1–8.1
Total	>120	72.9	70.5–75.3	>120	68.4	66.5–70.4	16.6	24.3	23.3–25.4	4.4	5.9	5.4–6.6

Notes: ^a^ Defined as per the Central Brain Tumour Registry of the United States. ^b^ NOS = not otherwise specified. ^c^ Values were suppressed due to one or more of the following reasons: the number of patients at risk was less than 50 and/or less than five deaths in the category. ^d^ Mean survival time is reported in months. ^e^ 5-year survival rates and associated 95% confidence intervals are presented as percentages.

**Table 4:** Median survival times (MSTs), five-year survival rates (5-year SRs), and associated 95% confidence intervals (CIs) for patients with primary non-malignant CNS tumours diagnosed between 2010–2017 in Canada (excluding Quebec) by histology and age group.

	Age Group (Years)											
	0 to 14			15 to 39			40 to 64			65+		
Histology Group ^a^ (Major/Specific)	MST ^d^	5-Year SR ^e^	95% CI	MST ^d^	5-Year SR ^e^	95% CI	MST ^d^	5-Year SR ^e^	95% CI	MST ^d^	5-Year SR ^e^	95% CI
**Tumours of neuroepithelial tissue**	>120	96.6	93.9–99.3	>120	95.1	92.7–97.5	>120	89.8	85.9–93.8	>120	71.4	61.3–83.2
Pilocytic astrocytoma												
Diffuse astrocytoma												
Anaplastic astrocytoma												
Unique astrocytoma variants ^c^	>120	95.0	85.9–100.0	>120	100	100.0–100.0	-	-	-	-	-	-
Glioblastoma												
Oligodendroglioma												
Anaplastic oligodendroglioma												
Oligoastrocytic tumours												
Ependymal tumours ^c^	-	-	-	>120	95.8	91.1–100.0	>120	93.2	88.8–97.7	>120	75.6	64.6–88.5
Glioma malignant, NOS ^b^												
Choroid plexus tumours ^c^	>120	100	100.0–100.0	-	-	-	>120	96.3	89.4–100.0	-	-	
Neuronal & mixed neuronal-glial tumours	>120	96.8	93.7–99.9	>120	94.3	91.3–97.4	113.2	82.3	74.2–91.2	75.5	57.1	36.9–88.5
Tumours of the pineal region	-	-	-	-	-	-	-	-	-	-	-	-
Embryonal tumours	-	-	-	-	-	-	-	-	-	-	-	-
Other neuroepithelial tumours	-	-	-	-	-	-	-	-	-	-	-	-
**Tumours of cranial & spinal nerves**	>120	100	100.0–100.0	>120	98.5	97.4–99.7	>120	96.9	95.9–97.8	>120	83.2	80.2–86.3
**Tumours of meninges**	>120	100	100.0–100.0	>120	97.1	95.8–98.3	>120	90.8	89.9–91.7	92.6	62.1	60.6–63.7
Meningioma	>120	100	100.0–100.0	>120	97.5	96.3–98.8	>120	90.9	90.0–91.8	90.8	61.7	60.1–63.3
Mesenchymal tumours ^c^	-	-	-	-	-	-	>120	89.4	82.1–97.4	98.4	74.3	60.8–90.7
Primary melanocytic lesions ^c^	-	-	-	-	-	-	-	-	-	-	-	-
Other neoplasms related to the meninges ^c^	-	-	-	>120	96.2	92.4–100.0	>120	90.3	85.8–95.0	>120	81.4	73.2–90.6
**Lymphomas & hematopoietic neoplasms**	-	-	-	-	-	-	-	-	-	-	-	-
Lymphoma												
Other hematopoietic neoplasms	-	-	-	-	-	-	-	-	-	-	-	-
**Germ cell tumours, cysts, & heterotopias ^c^**	>120	95.7	87.7–100.0	>120	100.0	100.0–100.0	>120	94.7	85.2–100.0	-	-	-
**Tumours of sellar region**	>120	93.7	88.4–99.3	>120	98.8	98.1–99.5	>120	94.7	93.7–95.7	>120	76.0	73.5–78.5
**Unclassified tumours**	>120	95.6	92.8–98.6	>120	93.3	91.4–95.2	>120	73.8	71.7–75.9	31.0	37.4	35.6–39.4
Total	>120	96.3	94.7–97.9	>120	96.9	96.3–97.5	>120	89.8	89.2–90.4	82.6	57.9	56.9–59.0

Notes: ^a^ Defined as per the Central Brain Tumour Registry of the United States. ^b^ NOS = not otherwise specified. ^c^ Values were suppressed due to one or more of the following reasons: the number of patients at risk was less than 50 and/or less than five deaths in the category. Histology groups that are 100% malignant are greyed out in the table. ^d^ Mean survival time is reported in months. ^e^ 5-year survival rates and associated 95% confidence intervals are presented as percentages.

**Table 5:** Person-based eight-year limited-duration prevalent cases (N), prevalence proportions (PPs, per 100,000 persons), and associated 95% confidence intervals (CIs) for primary malignant CNS tumours by histology and age group as of 1 January 2018.

	Age Group (Years)											
	0 to 14			15 to 39			40 to 64			65+		
Histology Group ^a^ (Major/Specific)	N	PP	95% CI	N	PP	95% CI	N	PP	95% CI	N	PP	95% CI
**Tumours of neuroepithelial tissue**	650	14.1	13.0–15.2	1355	14.3	13.5–15.1	2065	21.4	20.5–22.4	765	16.3	15.2–17.5
Pilocytic astrocytoma	215	4.7	4.1–5.3	225	2.4	2.1–2.7	70	0.7	0.6–0.9	5	0.1	0.03–0.3
Diffuse astrocytoma	35	0.8	0.5–1.1	175	1.8	1.6–2.1	170	1.8	1.5–2.1	30	0.6	0.4–0.9
Anaplastic astrocytoma	5	0.1	0.04–0.3	105	1.1	0.9–1.3	125	1.3	1.1–1.5	35	0.8	0.5–1.0
Unique astrocytoma variants ^b^	35 *	-	-	35 *	0.3	0.2–0.5	10	0.1	0.05–0.2	5	0.1	0.03–0.3
Glioblastoma	15	0.3	0.2–0.5	145	1.5	1.3–1.8	780	8.1	7.5–8.7	470	10.0	9.1–11.0
Oligodendroglioma ^b^	155 *	-	-	155 *	1.6	1.4–1.9	250	2.6	2.3–2.9	30	0.6	0.4–0.9
Anaplastic oligodendroglioma	0	-	-	60	0.6	0.5–0.8	170	1.8	1.5–2.1	30	0.6	0.4–0.9
Oligoastrocytic tumours ^b^	95 *	-	-	95 *	1.0	0.8–1.2	135	1.4	1.2–1.7	20	0.4	0.3–0.7
Ependymal tumours	80	1.7	1.4–2.2	80	0.8	0.7–1.1	135	1.4	1.2–1.7	60	1.3	1.0–1.7
Glioma malignant, NOS ^b^	100	2.2	1.8–2.6	130	1.4	1.1–1.6	140	1.5	1.2–1.7	50	1.1	0.8–1.4
Choroid plexus tumours	5	0.1	0.04–0.3	0	-	-	0	-	-	0	-	-
Neuronal & mixed neuronal-glial tumours ^c^	25 *	-	-	25 *	0.2	0.1–0.3	35	0.4	0.3–0.5	20	0.4	0.3–0.7
Tumours of the pineal region ^c^	10	0.2	0.1–0.4	15	0.2	0.09–0.3	10 *	0.1	0.05–0.2	10 *	-	-
Embryonal tumours ^c^	175	3.8	3.3–4.4	105	1.1	0.9–1.3	25 *	0.3	0.2–0.4	25 *	-	-
Other neuroepithelial tumours ^d^	10 **	-	-	10 **	-	-	10 **	-	-	10 **	-	-
**Tumours of cranial & spinal nerves**	0	-	-	10	0.05	0.02–0.1	5	0.05	0.02–0.1	5	0.1	0.03–0.3
**Tumours of meninges**	5	0.1	0.04–0.3	20	0.2	0.1–0.3	65	0.7	0.5–0.9	55	1.2	0.9–1.5
Meningioma ^c^	10 *	-	-	10 *	0.1	0.05–0.2	40	0.4	0.3–0.6	45	1.0	0.7–1.3
Mesenchymal tumours ^c^	10 *	-	-	10 *	0.1	0.05–0.2	20	0.2	0.1–0.3	5	0.1	0.03–0.3
Primary melanocytic lesions ^d^	10 **	-	-	10 **	-	-	10 **	-	-	10 **	-	-
Other neoplasms related to the meninges ^c^	10 *	-	-	10 *	-	-	10 *	0.05	0.02–0.1	10 *	-	-
**Lymphomas & hematopoietic neoplasms** ^c^	30 *	-	-	30 *	0.3	0.2–0.5	155	1.6	1.4–1.9	175	3.7	3.2–4.3
Lymphoma ^c^	25 *	-	-	25 *	0.3	0.2–0.4	150	1.6	1.3–1.8	175	3.7	3.2–4.3
Other hematopoietic neoplasms ^c^	10 *	-	-	10 *	-	-	10 *	-	-	10 *	-	-
**Germ cell tumours, cysts, & heterotopias**	30	0.7	0.4–0.9	90	1.0	0.8–1.2	10	0.1	0.05–0.2	0	-	-
**Tumours of sellar region ^c^**	10 *	-	-	10 *	-	-	10 *	-	-	5	0.1	0.03–0.3
**Unclassified tumours**	15	0.3	0.2–0.5	25	0.3	0.2–0.4	75	0.8	0.6–1.0	60	1.3	1.0–1.7
Total	700	15.1	14.0–16.3	1525	16.1	15.3–16.9	2375	24.6	23.7–25.6	1060	22.6	21.3–24.0

Columns and rows may not sum to totals due to rounding. ^a^ Defined as per the Central Brain Tumour Registry of the United States. ^b^ NOS = not otherwise specified. ^c^ Histology groups that have combined prevalent case counts across multiple age groups due to RDC vetting rules. Combined age group counts in each row are indicated by *. ^d^ Histology groups that have a combined prevalent case count that was rounded to 10 across all age groups and tumour behaviours (includes cases in the corresponding malignant histology group) are indicated by **.

**Table 6:** Person-based eight-year limited-duration prevalent cases (N), prevalence proportions (PPs, per 100,000 persons) and 95% confidence intervals (CIs) for primary non-malignant CNS tumours by histology and age group as of 1 January 2018.

	Age Group (years)											
	0 to 14			15 to 39			40 to 64			65+		
Histology Group ^a^ (Major/Specific)	N	PP	95% CI	N	PP	95% CI	N	PP	95% CI	N	PP	95% CI
**Tumours of neuroepithelial tissue**	120	2.6	2.2–3.1	430	4.5	4.1–5.0	310	3.2	2.9–3.6	95	2.0	1.6–2.5
Pilocytic astrocytoma												
Diffuse astrocytoma												
Anaplastic astrocytoma												
Unique astrocytoma variants ^d^	15	0.3	0.2–0.5	30	0.3	0.2–0.5	5 *	-	-	5 *	-	-
Glioblastoma												
Oligodendroglioma												
Anaplastic oligodendroglioma												
Oligoastrocytic tumours												
Ependymal tumours	5	0.1	0.04–0.3	85	0.9	0.7–1.1	155	1.6	1.4–1.9	60	1.3	1.0–1.7
Glioma malignant, NOS ^b^												
Choroid plexus tumours	25	0.5	0.4–0.5	20	0.2	0.1–0.3	25	0.3	0.2–0.4	10	0.2	0.1–0.4
Neuronal & mixed neuronal-glial tumours	75	1.6	1.3–2.0	280	3.0	2.6–3.3	115	1.2	1.0–1.4	25	0.5	0.3–0.8
Tumours of the pineal region	10 *	-	-	10 *	0.1	0.05–0.2	15 *	0.1	0.05–0.2	15 *	-	-
Embryonal tumours ^c^	10 *	-	-	10 *	-	-	10 *	-	-	0	-	-
Other neuroepithelial tumours ^d^	10 **	-	-	10 **	0.05	0.02–0.1	10 **	-	-	10 **	-	-
**Tumours of cranial & spinal nerves**	40	0.9	0.6–1.2	445	4.7	4.3–5.1	1630	16.9	16.1–17.8	940	20.0	18.8–21.4
**Tumours of meninges**	15	0.3	0.2–0.5	610	6.4	5.9–7.0	4435	46.0	44.7–47.4	4265	90.9	88.2–93.7
Meningioma	10	0.2	0.1–0.4	490	5.2	4.7–5.6	4140	42.9	41.6–44.3	4115	87.7	85.1–90.4
Mesenchymal tumours ^c^	10 *	-	-	10 *	0.1	0.05–0.2	60	0.6	0.5–0.8	40	0.9	0.6–1.2
Primary melanocytic lesions ^d^	10 **	-	-	10 **	-	-	10 **	0.05	0.02–0.1	10 **	-	-
Other neoplasms related to the meninges ^c^	105 *	-	-	105 *	1.1	0.9–1.3	225	2.3	2.0–2.7	110	2.3	1.9–2.8
**Lymphomas & hematopoietic neoplasms**	0	-	-	0	-	-	0	-	-	0	-	-
Lymphoma												
Other hematopoietic neoplasms	0	-	-	0	-	-	0	-	-	0	-	-
**Germ cell tumours, cysts, & heterotopias**	20	0.4	0.3–0.7	25	0.3	0.2–0.4	20	0.2	0.1–0.3	10	0.2	0.1–0.4
**Tumours of sellar region**	50	1.1	0.8–1.4	970	10.2	9.6–10.9	2335	24.2	23.3–25.2	1655	35.3	33.6–37.0
**Unclassified tumours**	165	3.6	3.1–4.2	795	8.4	7.8–9.0	1615	16.8	15.9–17.6	2080	44.3	42.5–46.3
Total	410	8.9	8.0–9.8	3275	34.5	33.3–35.7	10,340	107.3	105.2–109.3	9050	192.9	188.9–196.9

Columns and rows may not sum to totals due to rounding. ^a^ Defined as per the Central Brain Tumour Registry of the United States. ^b^ NOS = not otherwise specified. ^c^ Histology groups that have combined prevalent case counts across multiple age groups due to RDC vetting rules. Combined age group counts in each row are indicated by *. ^d^ Histology groups that have a combined prevalent case count rounded to 10 across all age groups and tumour behaviours (includes cases in the corresponding malignant histology group) are indicated by **. Histology groups that are 100% malignant are greyed out in the table.

## Data Availability

Death-linked Canadian Cancer Registry data is available through the Research Data Centres in Canada upon data access application approval from Statistics Canada.

## Supplementary Materials

The following supporting information can be downloaded at: https://www.mdpi.com/article/10.3390/curroncol30040329/s1, Figure S1: Age-standardized incidence rate of primary CNS tumours from 2008 to 2017 in Canada (excluding Quebec) by tumour behavior; Table S1: Proportion of unclassified primary CNS tumours diagnosed in Canada (excluding Quebec) between 2008 and 2017 by province and tumour behaviour.
