# Supplementary material for: The Incidence and Prevalence of Primary Central Nervous System (CNS) Tumours in Canada (2010–2017), and the Survival of Patients Diagnosed with CNS Tumours (2008–2017)
Source: Curr Oncol. 2023 Apr 20;30(4):4311–28. doi: 10.3390/curroncol30040329 (PMC10137065; doi:10.3390/curroncol30040329)
Supplement: Supplementary file 1 [file curroncol-30-00329-s001.zip › curroncol-2280749-supplementary.pdf]

Supplementary Figure S1: Age-standardized incidence rate of primary CNS tumours from 2008 to 2017 in Canada (excluding Quebec) by tumour behavior.

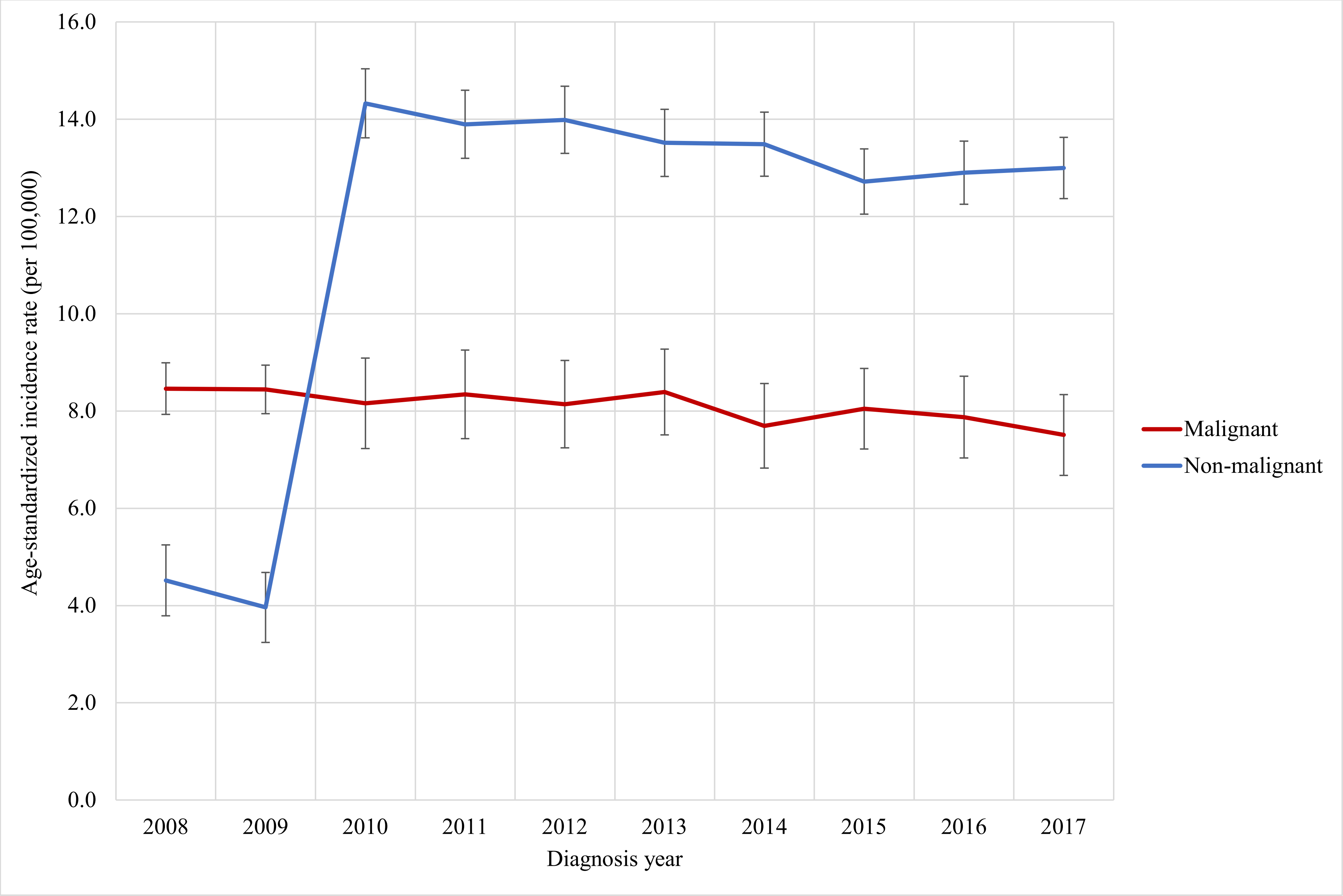

Supplementary Table S1: Proportion of unclassified primary CNS tumours diagnosed in Canada (excluding Quebec) between 2008 and 2017 by province and tumour behaviour.

<table>
<tr><th>Malignant</th><th>BC</th><th>AB</th><th>SK</th><th>MB</th><th>ON</th><th>NB</th><th>NS</th><th>NL</th></tr>
<tr><td>2008</td><td>0.046</td><td>0.018</td><td rowspan="10">–</td><td>0.063</td><td>0.174</td><td>0.083</td><td>0.048</td><td>0.100</td></tr>
<tr><td>2009</td><td>0.065</td><td>0.036</td><td>–</td><td>0.171</td><td>0.143</td><td>0.067</td><td rowspan="9">–</td></tr>
<tr><td>2010</td><td>0.059</td><td>0.021</td><td>0.063</td><td>0.094</td><td>–</td><td>0.053</td></tr>
<tr><td>2011</td><td>0.056</td><td>0.040</td><td>–</td><td>0.100</td><td>–</td><td>–</td></tr>
<tr><td>2012</td><td>0.027</td><td>0.040</td><td>–</td><td>0.087</td><td>–</td><td>–</td></tr>
<tr><td>2013</td><td>0.048</td><td>0.051</td><td>0.048</td><td>0.095</td><td>–</td><td>–</td></tr>
<tr><td>2014</td><td>0.079</td><td>0.036</td><td>0.059</td><td>0.082</td><td>–</td><td>–</td></tr>
<tr><td>2015</td><td>0.038</td><td>0.051</td><td>–</td><td>0.083</td><td>–</td><td>–</td></tr>
<tr><td>2016</td><td>0.064</td><td>0.017</td><td>–</td><td>0.080</td><td>–</td><td>0.059</td></tr>
<tr><td>2017</td><td>0.051</td><td>0.038</td><td>0.095</td><td>0.065</td><td>0.071</td><td>0.053</td></tr>
<tr><th>Non-malignant</th><th>BC</th><th>AB</th><th>SK</th><th>MB</th><th>ON</th><th>NB</th><th>NS</th><th>NL</th></tr>
<tr><td>2008</td><td>0.018</td><td>0.024</td><td>0.059</td><td>0.083</td><td>.</td><td rowspan="10">–</td><td rowspan="10">–</td><td rowspan="10">–</td></tr>
<tr><td>2009</td><td>0.036</td><td>0.027</td><td rowspan="9">–</td><td>0.031</td><td>.</td></tr>
<tr><td>2010</td><td>0.048</td><td>0.024</td><td>0.067</td><td>0.358</td></tr>
<tr><td>2011</td><td>0.034</td><td>0.036</td><td>0.075</td><td>0.341</td></tr>
<tr><td>2012</td><td>0.038</td><td>0.022</td><td>0.034</td><td>0.350</td></tr>
<tr><td>2013</td><td>0.029</td><td>0.022</td><td>0.031</td><td>0.368</td></tr>
<tr><td>2014</td><td>0.029</td><td>0.022</td><td>–</td><td>0.380</td></tr>
<tr><td>2015</td><td>0.032</td><td>0.033</td><td>0.037</td><td>0.379</td></tr>
<tr><td>2016</td><td>0.073</td><td>0.034</td><td>–</td><td>0.380</td></tr>
<tr><td>2017</td><td>0.056</td><td>0.039</td><td>–</td><td>0.427</td></tr>
</table>

Data sources: Canadian Cancer Registry at Statistics Canada
Province abbreviations are as follows: BC = British Columbia; AB = Alberta; SK = Saskatchewan; MB = Manitoba; ON = Ontario; NB = New Brunswick; NS = Nova Scotia; NL= Newfoundland & Labrador. – indicates number is suppressed. Numbers are suppressed in the Yukon and Prince Edward Island for all years and both behaviours. . indicates missing data.
